# A New Brief Measure of Oral Quality of Life

**Published:** 2008-03-15

**Authors:** Nancy R Kressin, Judith A Jones, Michelle B. Orner, Avron Spiro

**Affiliations:** Research Career Scientist, Center for Health Quality, Outcomes and Economic Research; Boston University School of Dental Medicine; Center for Health Quality, Outcomes and Economic Research, Veterans Administration Medical Center (VAMC), Bedford, Massachusetts; Center for Health Quality, Outcomes and Economic Research, VAMC, Bedford, Massachusetts; Center for Health Quality, Outcomes and Economic Research, VAMC, Bedford, Massachusetts; Normative Aging Study, Department of Veterans Affairs; and Boston Healthcare System, Boston University Goldman School of Dental Medicine, Boston University School of Public Health, Boston, Massachusetts

## Abstract

**Introduction:**

We developed a brief measure of the impact of oral conditions on individual functioning and well-being, known as oral quality of life.

**Methods:**

Among older male veterans (N = 827) and community dental patients (N = 113), we administered surveys consisting of extant oral quality of life items, using clinical dental data from the veteran samples. We assigned each oral quality of life item to a theoretical dimension, conducted an iterative series of multitrait scaling analyses to examine the item-fit with the dimensions, reduced the number of items, and examined the psychometric characteristics of new scales and their association with clinical indices.

**Results:**

We developed two brief oral quality of life scales, one consisting of 12 items and the other of 6, the latter a subset of the former. Each demonstrated sound psychometric properties and was sensitive to clinical indices.

**Conclusion:**

The two brief oral quality of life scales can be used to assess the population-based impact of oral conditions as well as outcomes of dental care.

## Introduction

The individual and public health impact of dental disease is increasingly recognized (1). However, dentistry has traditionally used specific clinical indices (e.g., number of teeth, periodontal attachment loss) to assess the impact of dental conditions. The limitations of using such clinical assessments of oral health status to understand the impact of oral disease are now clear (2): oral conditions affect the full scope of health status, including patients' functioning and well-being (e.g., oral quality of life [OQOL]) (3). Numerous patient-based measures of OQOL (4-8) have been developed, along with several clinician-based assessments (9-11). These measures vary in length (and, thus, ease of use in large-scale population-based surveys), in their sensitivity to clinical indices (or changes therein), and in their theoretical anchoring. Few studies have simultaneously examined the performance of items from more than one instrument (12).

Our goal was to produce a brief measure of oral health-related quality of life that was theoretically anchored and psychometrically and clinically valid, using the best-performing items from existing instruments, to provide a public health tool for assessing the individual and population impacts of oral health conditions.

## Methods

### Samples

We studied two groups of older male veterans from the Veterans Health Study and the Dental Longitudinal Study. The medical and oral health status of these men covered a broad range of conditions. We conducted a brief clinical oral exam (<15 minutes) and administered an oral health?related quality of life questionnaire to men in each study. In addition, to test our new brief instrument, we collected new questionnaire data on a sample of community dental patients at the time of a dental office visit. 

**1. The Veterans Health Study (VHS)**, begun in 1993, is a large-scale observational study (N = 2425) of veterans (mean age at the time of the study, 62 years), who are representative of independent, community-dwelling veterans who use Department of Veterans Affairs (VA) ambulatory care; as such, they had a variety of chronic medical conditions (13,14). A subset of 538 men completed the OQOL measures as part of an auxiliary study (15).

**2. The Dental Longitudinal Study (DLS)** is the dental component consisting of 1231 participants from the VA Normative Aging Study (NAS), a closed?panel longitudinal study of aging begun in 1968 among 2280 community-dwelling male veterans (mean age at the time of the study, 67 years) (16,17). The DLS is focused on oral health in aging men (18). Most are veterans but are *not* VA patients and are generally representative of the adult male population in the greater Boston metropolitan area, although they may be healthier, because all subjects were required to be medically healthy for entry into the NAS. DLS participants receive clinical dental exams triennially and were asked to complete an additional questionnaire concerning their oral quality of life; 289 participated.

**3. Community dental patients **who were visiting one of 16 community dental offices for prophylaxis, endodontic care, or placement of a removable prosthesis were provided our OQOL survey; 113 patients, both male and female, participated.

Data collection from each sample was approved by the local institution's institutional review board, and all participants gave written informed consent.

### Theoretical framework

Our work was guided by a broad conceptualization of health and quality of life widely used in the general and oral health-related quality of life literature, which includes five broad domains (19-22):

- *Survival* is equated to mortality or longevity of the tooth or orofacial structures.
- *Impairments and disease* (or illness) include symptoms and indications of discomfort or pain.
- *Functional states* include three domains: a) *physical* functioning (e.g., activity restrictions, difficulties eating, chewing, or speaking); b) *social* functioning (e.g., the ability to perform social roles such as speaking, smiling, eating in public, being able to meet work and family obligations); and c) *psychological* functioning (e.g., patient satisfaction with the aesthetics of their dentition; comfort with interpersonal relations; worry, concern, embarrassment about, or lack of confidence caused by problems with teeth or gums).
- Oral health *perceptions* include one's global assessment of, and satisfaction with, oral health status and aesthetics, including need for treatment.
- Finally, *opportunity and resilience* reflect disadvantages incurred as a result of oral health and the impact of the disadvantages on one's ability to function in social and work roles and to have good nutrition through a satisfactory ability to eat and chew.

We adapted this model to fit an oral/dental framework and then examined how well the adapted model fit our data on oral health and quality of life. We hypothesized a framework with four primary dimensions: 1) physical function, 2) psychosocial functioning (with three subdimensions: role function, distress, and worry), 3) impairment or disease, and 4) perceptions.

### Measures

The survey that we administered to our two veteran study populations included three extant OQOL measures (the Geriatric Oral Health Assessment Instrument, the Oral Health-Related Quality of Life [OHQOL] measure, and the Oral Health Impact Profile) with a total of 64 OQOL items, concurrent with a clinical oral exam. The community dental patient sample received a survey with an abbreviated selection of OQOL items but no clinical dental exam.

The Geriatric Oral Health Assessment Instrument (GOHAI) (5) comprises 12 items reflecting 3 domains of impact of oral disease: physical function, psychosocial function, and pain or discomfort.

The OHQOL measure is a brief global assessment of the impact of oral conditions on an individual's functioning and well-being (4). The three OHQOL items assess the extent to which problems with teeth or gums influence an individual's daily activities, social interactions, or avoidance of conversations.

Slade and Spencer (23) developed the Oral Health Impact Profile (OHIP), an empirically grounded 49-item instrument based on a conceptual framework of oral disease and its functional and psychosocial consequences. The OHIP contains seven subscales: 1) functional limitation, 2) pain, 3) psychological discomfort, 4) physical disability, 5) psychological disability, 6) social disability, and 7) disadvantage.

### Clinician-assessed oral health status

We collected clinical data in the VHS and DLS. To assess periodontal treatment need, we used the Community Periodontal Index of Treatment Need (CPITN) (24-26), which is based on measures taken from 10 teeth from the 6 sextants of the mouth, yielding an index score (ordinal scale of 0–4). This index was developed by the World Health Organization as an efficient measure for use in epidemiologic studies of periodontal status and treatment needs. *Coronal caries* and restorations were scored as in protocols of the National Institute of Dental Research for its National Survey of Oral Health in Adults (27), whereas* root caries* measures used an index developed by Hayes and Katz (28). This latter methodology, which has been used in two large epidemiologic studies, is efficient because it requires assessment of root caries and restorations on only eight tooth surfaces instead of every tooth in the mouth (29,30).

### Procedure and data analysis

First, three of the authors (NK, JJ, AS) independently categorized each item from the three OQOL instruments into one of the theoretical domains described above. Any differences in domain assignments were resolved by consensus. Next, using existing data from the two veteran samples (N = 827), we conducted a series of psychometric analyses and examined the fit of the items to the hypothesized domains, using numerous iterations of multitrait scaling analysis (30,31), which is built on the logic of the multitrait-multimethod approach (32). Multitrait scaling analyses examine item-level characteristics (e.g., amount of missing data, frequency distribution, mean, standard deviation), the relationship of each item to other items in the scale that the item is hypothesized to measure, as well as the item's relationship to other scales. This analytic method provides information about scale distribution characteristics (e.g., mean, standard deviation, range, percentage of respondents scoring at the floor and ceiling) as well as the reliability of the scale scores and correlations among hypothesized scales. Compared with exploratory factor analysis, another commonly used approach to scale development, multitrait scaling analyses take a more confirmatory approach, evaluating the appropriateness of a priori groupings of items, allowing the investigator to specify and analyze conceptually meaningful groups of items. Item internal consistency (the extent to which the item is related to the concept being measured) is considered acceptable if an item correlates 0.40 or more with its hypothesized scale, after correction for item-scale overlap (30).

Item discriminant validity (the extent to which the item measures what it is supposed to measure) is considered acceptable if the correlation between the item and its hypothesized scale is significantly higher than the item's correlation with all other scales (32); we used the significance level of two standard errors (95% confidence interval) for this criterion. For internal consistency reliability (the extent to which items within a scale share common variance), we considered a Cronbach ? of .70 to be acceptable (33).

Multiple approaches may be used to produce short-form measures of health-related quality of life (15), including item impact studies, factor analytic approaches (described above), and stepwise regression analysis. We were unable to adopt an item impact approach because we did not have item impact data for two of the three measures we used, and our choice of the multitrait scaling analysis was largely driven by our desire to confirm and refine our hypothesized conceptual schema.

## Results

On the basis of the initial multitrait scaling analyses, we identified three items (GOHAI3, GOHAI5, GOHAI8)On the basis of the initial multitrait scaling analyses, we identified three items (GOHAI3, GOHAI5, GOHAI8) that correlated poorly with all of the domains we originally hypothesized, so we eliminated these items. We created a separate denture subscale, recognizing that denture functioning represents a conceptual dimension separate from that of natural teeth; this also further improved scaling properties. Results indicated that the psychosocial and opportunity items covered four dimensions: 1) distress, 2) self-consciousness and worry, 3) role function, and 4) opportunity. Because most of the items in the latter construct loaded more strongly on other scales and because of skepticism about the usefulness of opportunity as an oral health construct, we deleted these three items (OHIP29, OHIP45, OHIP47). Thus, we were left with three remaining psychosocial constructs: distress, self-consciousness and worry, and role function. Additional analyses found that some items had poor loadings on the hypothesized dimensions. Accordingly, we moved the perception items (OHIP44, OHIP3, GOHAI7) from the perception dimension into the worry dimension of psychosocial items, where they had higher loadings.

We then examined the impairment items, using exploratory factor analysis, because of concerns about the multidimensionality of this domain. Indeed, we found four subdimensions: 1) mouth pain, 2) flavor, digestion, and breath, 3) tooth pain, and 4) denture discomfort. On the basis of these results, we retained all of these items but further altered our conceptual model to include five dimensions: 1) physical function, 2) impairment and disease, and three dimensions of psychosocial function: 3) role function, 4) distress, and 5) worry (Appendix 1).  

Using the remaining items, we standardized the item scores so that the mean of each variable was 50 and the standard deviation, 10. We scored the scales by taking the mean of all the items, after reversing the response categories where necessary so that higher scores indicated poorer oral quality of life. Thus, we created five scales to correspond with the above dimensions, a separate scale of the three denture-related items, and a summary scale comprising all items.

To develop a shorter version of the measure, we used data from the two veteran samples analyzed together to conduct forward stepwise regressions on each scale. This process allowed us to determine which items explained the most variance in each scale score. We selected items that explained either 80% of the variance or the first five items, whichever was greater. This resulted in five scales, each with five items. All of the scales had excellent internal consistency reliability, ranging from .78 (impairment) to .92 (distress), with the other scales also having excellent reliability (Table 1).

Next, we examined the correlations of each scale with clinical indices (Table 1). The strongest correlation observed was between physical function and number of teeth (*r* = −0.38). Coronal caries was moderately associated with worry (*r* = 0.23) and impairment (*r* = 0.18), whereas periodontal status was moderately associated with physical function (*r *= 0.21) and worry (*r *= 0.21). Root caries had the smallest correlations overall with OQOL.

We also examined mean scores on each of the quality of life dimensions by scores on the CPITN and found that individuals with greater treatment need had significantly worse OQOL (Table 2).

We then examined the proportion of variance explained in each oral quality of life dimension among different subgroups based on number of teeth (not shown). The impairment, physical function, worry, and role function scales explained the least variance among patients with no teeth and the most among patients having 1 to 10 teeth. The patterns observed for the distress scale were different: the most variance was explained among those with either no teeth or 1 to 10 teeth, with the least (but still 97% of the variance explained) among those with 11 to 24 teeth.

Next, we administered these five scales and the three denture-specific items to the sample of community dental patients. Using multitrait analysis, we sought to reduce the number of items further by eliminating items contributing least to each scale's internal consistency reliability and retaining items that conceptually best represented the spirit of the subscale. We eliminated items whose deletion least affected the internal consistency reliability of the scales (Cronbach α), and at the same time, sought to retain the items that we considered, from a conceptual standpoint, best represented the spirit of the subscale. We did this on two levels. First, we developed one 12-item measure (Appendix 2) that includes 3-item subscales for each of 3 scales in the psychosocial dimension (distress, worry, and role) and single items assessing dimensions entitled *physical, denture,* and *pain* (Cronbach α of the 12 items = .90). We also developed a second, briefer 6-item measure that includes single items assessing each dimension (distress, worry, role, physical, denture, pain) (Cronbach α for the scale = .80). 

We then took these two brief measures, refined on the community dental patient sample, and returned to our original data set of 827 veterans to examine the association of the two brief scales with clinical indices. Both summary scales were significantly correlated overall with number of teeth (*r* = −0.35 and −0.23, for the 6- and 12-item scales, respectively), coronal decay (*r* = 0.09 and 0.14), periodontal status (*r* = 0.19 and 0.20), and root caries (*r* = 0.14 and 0.12) (Table 3). Most items were significantly correlated with number of teeth, coronal decay, and periodontal status, but fewer were significantly correlated with root caries. Most items were associated with periodontal treatment need (Table 4).

## Discussion

We sought to develop a brief oral quality of life measure that is theoretically anchored, psychometrically sound, and clinically responsive from items comprising three existing OQOL indices, and that can be used by public health researchers, practitioners, and policy makers to assess the impact of oral conditions on people's functioning and well-being. We conducted extensive psychometric analyses, reducing the original 64-item pool to a set of 25 items comprising 5 theoretically derived scales that demonstrate sound psychometric properties and associations in the expected directions with clinical indices. We further reduced the number of items to two brief scales (one with 12 items and one with 6 items) that maintained strong psychometric characteristics. Both scales were sensitive to differences in clinical dental status, supporting their validity.

Taken together, the findings indicate that these new oral quality of life measures are sensitive to clinical indicators of oral health status, suggesting their usefulness in monitoring population health, for making prevalence estimates, for monitoring secular trends in population changes, and for studying the effects of public health interventions designed to prevent or reduce the effects of oral disease. The associations we detected between the oral quality of life measure and clinical indices are similar to those of other published findings (12,36).

The intermediate results of examining the properties of the five 5-item scales showed that each scale accounted for a suitably high proportion of the variance. Thus, we  conclude that the conceptual domains are well represented by the items in each scale. Importantly, our results indicate that both short-form scales are also sensitive to differences in clinical status and would be feasible to use in the clinical setting as an outcomes measure or in the general population to assess the impact of differing clinical status. The observed differences in internal consistency reliability suggest that the 6-item measure is appropriate for comparing groups of people, whereas the 12-item measure would be appropriate for assessing outcomes among individuals.

These results were limited by our partial reliance on cohorts of veterans who were older and all men, and who are thus not representative of the population as a whole. However, this disadvantage was offset by the availability of a rich clinical data set on these cohorts. Furthermore, the absence of detailed sociodemographic data on the community dental sample limited our ability to examine associations with these factors.

What value does this new brief OQOL instrument add to the literature, especially given that there are other OQOL instruments of similar length (e.g., OHIP14, GOHAI)? Other authors have compared the performance of various OQOL measures in their entirety (e.g., the OHIP14 and the OIDP [36,37] or the GOHAI and OHIP14 [38]), but to our knowledge, none have evaluated the relative performance of individual items from multiple OQOL measures. Our new measure has undergone extensive psychometric analysis and evaluation regarding its sensitivity to clinical indices and, although others have conducted similar types of analyses (36,38) using less detailed clinical data, the availability of extensive clinical dental data on our cohorts provides added confidence in this measure's sensitivity to differences in oral health status. Slade (38) examined the relative performance of the 49 original OHIP items and developed a 14-item short form of the OHIP. Two of the items in his short-form measure also are present in ours (finding it difficult to relax with oral problems and being totally unable to function because of oral problems), but several other OHIP items in our measure did not survive his item-reduction process, and 12 items in his short-form measure did not survive our item-reduction efforts.

Efforts are under way to use these new measures among populations of dental patients (39), including evaluations of responsiveness to change in clinical status (40). Future research would also benefit from a comparison of the performance of this new brief measure to that of other OQOL measures of similar length, in multiple settings, including the community, as well as in private dental offices, or as a treatment outcomes measure for use by dental insurers.

## Figures and Tables

**Table 1:** Correlations of Scales (5 Items in Each) With Clinical Variables, Scale Internal Consistency Reliability, and Variance Explained by Each Scale, Among Participants in the Veterans Health Study and the Dental Longitudinal Study, 1993–1995 (N = 827)^a^

Scale	No. of Teeth **(*P*)**	Coronal Caries^b^ **(*P*)**	Periodontal Status^c^ **(*P*)**	Root Caries^d^ **(*P*)**	Cronbach α	Variance Explained
Impairment	−0.02^e^ (0.55)	0.18 (<.001)	0.19 (<.001)	0.13 (.004)	0.78	0.91
Physical	−0.38 (<.001)	0.10 (.011)	0.21 (<.001)	0.14 (.002)	0.81	0.94
Distress	−0.16 (<.001)	0.11 (.005)	0.14 (.002)	0.08^e^ (.11)	0.92	0.97
Worry	−0.14 (<.001)	0.23 (<.001)	0.21 (<.001)	0.15 (.001)	0.84	0.93
Role	−0.19 (<.001)	0.08 (.02)	0.16 (<.001)	0.08^e^ (.11)	0.86	0.96
Denture	−0.57 (<.001)	−0.09 (.01)	0.09^e^ (.06)	0.09^e^ (.05)	NA	NA
**Summary scale**	−0.21 (<.001)	0.16 (<.001)	0.21 (<.001)	0.13 (.01)	NA	NA

NA indicates not applicable.

a Pearson correlation coefficients were used to obtain means. Higher oral quality of life scores represent poorer quality of life.

b Coronal caries indicates coronal decayed surfaces at level 2 or greater.

c Periodontal status indicates per person mean Community Periodontal Index of Treatment Need (CPITN) score of available sextants.

d Root caries indicates mean percentage of exposed root surfaces with unfilled decay.

e Correlations are not statistically significant.

**Table 2:** Mean^a^ Oral Quality of Life Scores by Varying Levels of Periodontal Disease Among Participants in the Veterans Health Study and the Dental Longitudinal Study, 1993–1995 (N = 827)

Scale	CPITN Score^b^			

	<1	1-1.9	2-2.9	≥3
All	48.0 (a)	47.4 (a)	51.4 (b)	53.9 (b)
Impairment	48.7 (a,b)	48.1 (a)	51.9 (b)	55.4 (c)
Physical	46.6 (a)	46.5 (a)	50.4 (b)	53.3 (b)
Distress	49.0 (a,b)	48.2 (a)	50.8 (a,b)	51.8 (b)
Worry	47.7 (a)	47.8 (a)	52.0 (b)	54.2 (b)
Role	49.2 (a,b)	47.9 (a)	50.9 (a,b)	52.7 (b)

CPITN indicates Community Periodontal Index of Treatment Need.

a Means were obtained from analysis of variance (ANOVA) testing and compared by using Duncan's multiple range test (35). Different letters indicate groups are significantly different from one another at *P* <.05; if the same letter is present, the groups are not different from one another. Thus, a mean labeled (a,b) is not significantly different from one labeled (b,c) because they both have a "b" beside them. Higher oral quality of life scores represent poorer quality of life.

b CPITN scores are as follows: <1, healthy periodontium; 1-1.9, gingival bleeding; 2-2.9, calculus; ≥3, moderate-deep periodontal pockets (need root planing).

**Table 3:** Correlations^a^ of Items From the Two New Brief Scales and Overall Summary Scales With Clinical Variables Among Participants in the Veterans Health Study and the Dental Longitudinal Study, 1993–1995 (N = 827)

Scale	No. of Teeth** (*P*)**	Coronal Caries^b^ **(*P*)**	Periodontal Status^c^ **(*P*)**	Root Caries^d^ **(*P*)**	Scale/Item No.
**Impairment**					
Past 3 months how much pain and distress^e^	−0.11 (.003)	0.11 (.004)	0.15 (<.001)	0.12 (.01)	OHQOL B31
**Physical**					
** **Have to avoid eating any food	−0.35 (<.001)	0.07^f^ (.06)	0.17 (<.001)	0.14 (.004)	OHIP 28
**Distress**					
Found difficult to relax with oral problems	−0.13 (<.001)	0.09 (.01)	0.14 (.003)	0.08^f^ (.08)	OHIP 35
Feel depressed with oral problems	−0.16 (<.001)	0.10 (.009)	0.13 (.005)	0.06^f^ (.18)	OHIP 36
Being upset with oral problems	−0.12 (.001)	0.08 (.04)	0.12 (.01)	0.06^f^ (.17)	OHIP 34
**Worry**					
Uncomfortable about oral appearance	−0.12 (.001)	0.20 (<.001)	0.20 (<.001)	0.11 (.02)	OHIP 22
Past 3 months feel nervous or self-conscious—teeth^e^	−0.15 (<.001)	0.14 (<.001)	0.18 (<.001)	0.09^f^ (.08)	GOHAI 10
Worried by dental problems	−0.06^f^ (.13)	0.21 (<.001)	0.17 (<.001)	0.12 (.008)	OHIP 19
**Role**					
Not get along with others	−0.13 (<.001)	0.08 (.04)	0.12 (.008)	0.05 (.27)	OHIP 41
Avoid going out with oral problems	−0.14 (<.001)	0.11 (.004)	0.17 (<.001)	0.06 (.20)	OHIP 39
Totally unable to function with oral problems	−0.11 (.003)	0.05 (.16)	0.11 (.02)	0.09 (.06)	OHIP 48
**Denture**					
Have uncomfortable dentures	−0.53 (<.001)	−0.08 (.03)	0.05 (.28)	0.07 (.11)	OHIP 18
**Summary scale (6 items)**	−0.35 (<.001)	0.09 (.009)	0.19 (<.001)	0.14 (.003)	NA
**Summary scale (12 items)**	−0.23 (<.001)	0.14 (<.001)	0.20 (<.001)	0.12 (.01)	NA

OHIP indicates Oral Health Impact Profile; GOHAI indicates Geriatric Oral Health Assessment Instrument; OHQOL indicates Oral Health Quality of Life; NA indicates not applicable.

a Pearson correlation coefficients were used to obtain means. Higher oral quality of life scores represent poorer quality of life.

b Coronal caries indicates coronal decayed surfaces at level 2 or greater.

c Periodontal status indicates per person mean Community Periodontal Index of Treatment Need (CPITN) score of available sextants.

d Root caries indicates mean percentage of exposed root surfaces with unfilled decay.

e Scores were reversed so that higher scores indicate poorer oral quality of life.

f Correlations are not statistically significant.

**Table 4:** Mean Oral Quality of Life Scores^a^ by Varying Levels of Periodontal Treatment Need Among Participants in the Veterans Health Study and the Dental Longitudinal Study, 1993–1995 (N = 827)

**Variables**	CPITN Score^b^					Scale/Item No.

	≤1	1-1.9	2-2.9	3-3.9	*P *value	
**Impairment**						
Past 3 months how much pain and distress^c^	16.7 (b)	15.2 (b)	23.3 (a,b)	25.7 (a)	.003	OHQOL B31
**Physical**						
Have to avoid eating any food	13.9 (c)	15.9 (b,c)	23.4 (b)	34.1 (a)	<.001	OHIP 28
**Distress**						
Found difficult to relax with oral problems	15.6 (a)	13.8 (a)	19.9 (a)	21.6 (a)	.04	OHIP 35
Feel depressed with oral problems	17.0 (a)	13.8 (a)	21.9 (a)	21.0 (a)	.019	OHIP 36
Being upset with oral problems	18.3 (a)	14.2 (a)	19.5 (a)	21.6 (a)	.124	OHIP 34
**Worry**						
Uncomfortable about oral appearance	15.7 (b)	16.1 (b)	28.0 (a)	27.8 (a)	<.001	OHIP 22
Past 3 months feel nervous or self-conscious — teeth^c^	8.9 (b)	10.2 (b)	20.2 (a)	23.2 (a)	.001	GOHAI 10
Worried by dental problems	23.5 (b,c)	22.5 (c)	32.3 (a,b)	39.2 (a)	<.001	OHIP 19
**Role**						
Cannot get along with others	8.3 (a)	8.4 (a)	11.2 (a)	14.8 (a)	.16	OHIP 41
Avoid going out with oral problems	7.9 (a,b)	5.4 (b)	12.5 (a)	14.2 (a)	.003	OHIP 39
Totally unable to function with oral problem	6.9 (a,b)	5.1 (b)	7.8 (a,b)	11.9 (a)	.07	OHIP 48
**Denture**						
Have uncomfortable dentures	8.3 (a)	5.3 (a)	12.1 (a)	12.6 (a)	.02	OHIP 18
**6-item scale**	12.4 (b)	10.8 (b)	18.4 (a)	21.8 (a)	<.001	NA
**12-item scale**	13.4 (b,c)	11.8 (c)	18.9 (a,b)	21.2 (a)	<.001	NA

CPITN indicates Community Periodontal Index of Treatment Needs; OHIP indicates Oral Health Impact Profile; GOHAI indicates Geriatric Oral Health Assessment Instrument; OHQOL indicates Oral Health Quality of Life; NA indicates not applicable.

a Means were obtained from analysis of variance (ANOVA) testing and compared by using Duncan's multiple range test (35). Different letters indicate groups are significantly different from one another at *P* <.05; if the same letter is present, the groups are not different from one another. Thus, a mean labeled (a,b) is not significantly different from one labeled (b,c) because they both have a 'b' beside them. Higher oral quality of life scores represent poorer quality of life.

b CPITN scores are the following: <1, healthy periodontium; 1–1.9, gingival bleeding; 2–2.9, calculus; 3-3.9, moderate-deep periodontal pockets (need root planing).

c Scores were reversed so that higher scores indicate poorer oral quality of life.

## Appendices

### Appendix 1. Items Comprising Each 5-Item Oral Quality of Life Scale and the Denture Scale

#### Impairment

1. Have you had painful aching in your mouth? (OHIP9)
2. Have you felt that your sense of taste has worsened? (OHIP6)
3. Have you had sensitive teeth, for example, due to hot or cold foods or drinks? (OHIP12)
4. How much pain or distress have your teeth or gums caused you? (OHQOL B31)
5. Have you felt that your breath has been stale? (OHIP5)

#### Physical

1. Have you had to avoid eating some foods? (OHIP28)
2. Have you had trouble pronouncing any words? (OHIP2)
3. How often did you limit the kinds or amounts of food you eat because of problems with your teeth or dentures? (GOHAI1)
4. How often have problems with your teeth and gums affected your daily activities (such as work or hobbies)? (OHQOL1)
5. Have you found it uncomfortable to eat any foods? (OHIP15)

#### Distress

1. Have you found it difficult to relax? (OHIP35)
2. Have you felt depressed? (OHIP36)
3. Have you been a bit irritable with other people? (OHIP42)
4. Have you been upset? (OHIP34)
5. Have you been unable to enjoy other people's company as much? (OHIP46)

#### Worry

1. Have you felt uncomfortable about the appearance of your teeth, mouth or dentures? (OHIP22)
2. How often did you feel nervous or self-conscious because of problems with your teeth, gums, or dentures? (GOHAI10)
3. How often have problems with your teeth and gums affected your social activities (such as with family, friends, coworkers)? (OHQOL2)
4. Have you avoided smiling? (OHIP31)
5. Have you been worried by dental problems? (OHIP19)

#### Role

1. Have you had trouble getting along with other people? (OHIP41)
2. Have you been unable to work to your full capacity? (OHIP49)
3. Have you avoided going out? (OHIP39)
4. Have you been totally unable to function? (OHIP48)
5. Have people misunderstood some of your words? (OHIP25)

#### Denture

1. Have you felt that your dentures have not been fitting properly? (OHIP17)
2. Have you had uncomfortable dentures? (OHIP18) 
3. Have you been unable to eat with your dentures because of problems with them? (OHIP30)

OHIP indicates Oral Health Impact Profile; GOHAI, Geriatric Oral Health Assessment Instrument; OHQOL, Oral Health Quality of Life.

### Appendix 2. Short Form 12-Item Oral Quality of Life Measure

**Table:**

**During the past 3 months** HOW OFTEN have you experienced the following difficulties because of problems with your teeth, mouth, or dentures? (Circle one answer)	**Never**	**Hardly Ever**	**Occasionally**	**Fairly Often**	**Very Often**
*1. Have you had to avoid eating some foods? (OHIP28)	0	1	2	3	4
*2. Have you found it difficult to relax? (OHIP35)	0	1	2	3	4
3. Have you felt depressed? (OHIP36)	0	1	2	3	4
4. Have you been upset? (OHIP34)	0	1	2	3	4
5. Have you felt uncomfortable about the appearance of your teeth, mouth, or dentures? (OHIP22)	0	1	2	3	4
6. Have you been worried by dental problems? (OHIP19)	0	1	2	3	4
7. Have you had trouble getting along with other people? (OHIP41)	0	1	2	3	4
*8. Have you avoided going out? (OHIP39)	0	1	2	3	4
9. Have you been totally unable to function? (OHIP48)	0	1	2	3	4

**Table:**

**In the past 3 months**, how often:	**Never**	**Sometimes**	**Always**
*10. Did you feel nervous or self-conscious because of problems with your teeth, gums, or dentures? (GOHAI10)	1	2	3

**Table:**

*11. **During the past 3 months**, how much pain or distress have your teeth or gums caused you? (OHQOL B31)	**None at All**	**A Little Bit**	**Some**	**Quite a Bit**	**A Great Deal**
	1	2	3	4	5
If you have removable denture appliances, please answer the following question:					
**During the past 3 months**, how often have you had the following problems with your dentures?	**Never**	**Hardly Ever**	**Occasionally**	**Fairly Often**	**Very Often**
*12. Have you had uncomfortable dentures? (OHIP18)	0	1	3	4	5

OHIP indicates Oral Health Impact Profile; GOHAI, Geriatric Oral Health Assessment Instrument; OHQOL, Oral Health Quality of Life.

* Indicates items included in 6-item measure.
